# The Potential Regulatory Mechanisms of miR-196a in Huntington’s Disease through Bioinformatic Analyses

**DOI:** 10.1371/journal.pone.0137637

**Published:** 2015-09-16

**Authors:** Mu-Hui Fu, Chia-Ling Li, Hsiu-Lien Lin, Shaw-Jeng Tsai, Yen-Yu Lai, Yu-Fan Chang, Pei-Hsun Cheng, Chuan-Mu Chen, Shang-Hsun Yang

**Affiliations:** 1 Institute of Basic Medical Sciences, College of Medicine, National Cheng Kung University, Tainan 70101, Taiwan; 2 Department of Physiology, College of Medicine, National Cheng Kung University, Tainan 70101, Taiwan; 3 Department of Neurology, Kaohsiung Chang Gung Memorial Hospital and Chang Gung University College of Medicine, Kaohsiung, Taiwan; 4 Division of Breeding and Genetics, Livestock Research Institute, Council of Agriculture, Tainan 71246, Taiwan; 5 Department of Life Sciences, Agricultural Biotechnology Center, National Chung Hsing University, Taichung 40227, Taiwan; University of Alabama at Birmingham, UNITED STATES

## Abstract

High throughput screening is a powerful tool to identify the potential candidate molecules involved during disease progression. However, analysis of complicated data is one of the most challenging steps on the way to obtaining useful results from this approach. Previously, we showed that a specific miRNA, miR-196a, could ameliorate the pathological phenotypes of Huntington’s disease (HD) in different models, and performed high throughput screening by using the striatum of transgenic mice. In this study, we further tried to identify the potential regulatory mechanisms using different bioinformatic tools, including Database for Annotation, Visualization and Integrated Discovery (DAVID), Molecular Signatures Database (MSigDB), TargetScan and MetaCore. The results showed that miR-196a dominantly altered “ABC transporters”, “RIG-I-like receptor signaling pathway”, immune system”, “adaptive immune system”,“tissue remodeling and wound repair” and “cytoskeleton remodeling”. In addition, miR-196a also changed the expression of several well-defined pathways of HD, such as apoptosis and cell adhesion. Since these analyses showed the regulatory pathways are highly related to the modification of the cytoskeleton, we further confirmed that miR-196a could enhance the neurite outgrowth in neuroblastoma cells, suggesting miR-196a might provide beneficial functions through the alteration of cytoskeleton structures. Since impairment of the cytoskeleton has been reported in several neuronal diseases, this study will provide not only the potential working mechanisms of miR-196a but also insights for therapeutic strategies for use with different neuronal diseases.

## Introduction

Huntington’s disease (HD) is a dominantly inherited neurodegenerative disease caused by an expansion of CAG repeats located in first exon of the *Huntingtin* (*HTT*) gene. It is characterized by neuropathological changes in brain regions, such as the cortex and striatum. Among these, striatal medium spiny neurons are the most vulnerable [1, 2]. Various cell and animal models of HD indicate that the proteolysis of full-length HTT generates a number of small N-terminal HTT fragments, which are misfolded into aggregates in soma and neurites [3, 4]. HD patients typically develop impairment of motor functions, and one of the most common clinical symptoms of HD is chorea, an abnormal involuntary movement disorder with severe voluntary and goal-directed motor dysfunctions [5, 6]. To date, no cure for HD is available.

Recently, several researchers have focused on therapy to delay the progression of HD. RNA interference (RNAi) is one promising approach, and it can suppress the expression of mutant HTT at the post-transcriptional level [7–9]. microRNA is one of the RNAi regulatory pathways, and downregulates gene expression by binding to complementary sites in the 3’ untranslated region (3’UTR) of target mRNAs, further inhibiting protein translation [10, 11]. Previous studies indicated that miRNAs were responsible for neuronal development and involved in several neurodegenerative processes [12, 13]. Furthermore, miRNAs are known to regulate disease progression in HD [14–16]. These results suggest that miRNAs should play an important role in HD, and may be considered for use as a therapeutic strategy in the future.

According to our previous study, a specific miRNA, miR-196a, could ameliorate the HD phenotypes in cell, transgenic mouse and induced pluripotent stem cell models [14], suggesting that it regulates endogenous pathways to improve HD. To further comprehensively investigate the affected pathways of gene regulation, we performed high-throughput mRNA microarray (GSE47500) by using striatal tissues from HD transgenic mice and HD transgenic mice overexpressing miR-196a. We have now identified several critical pathways influenced by miR-196a in HD using a bioinformatic approach, and provided new insights with regard to the protective mechanisms of miR-196a in HD.

## Materials and Methods

### Microarray analysis

Two HD transgenic mice and three HD transgenic mice overexpressing miR-196a at approximately 12 months of age were used for this microarray analysis. At this stage, HD transgenic mice showed severe motor dysfunctions, whereas HD transgenic mice overexpressing miR-196a displayed mild motor dysfunctions[14]. RNAs were extracted from the striatum regions of two transgenic mouse lines, and then subjected to microarray analysis using the Mouse Whole Genome OneArray (Phalanx Biotech Group). The technique was carried out twice for each sample. Raw data were uploaded to Gene Expression Omnibus (GEO), and the accession number is GSE47500.

### Bioinformatic analysis

Four bioinformatics tools were used in this study. Functional annotation and biological category enrichment were imported into MetaCore (GeneGo, http://multicourse.binfo.ncku.edu.tw/genego2/all.php), DAVID (Database for Annotation, Visualization and Integrated Discovery, http://david.abcc.ncifcrf.gov) and MSigDB (Molecular Signatures Database, http://www.broadinstitute.org/gsea/index.jsp). The predicted target genes of miR-196a were obtained via TargetScan 6.2 (http://www.targetscan.org/).

### Neurite outgrowth

N2a mouse neuroblastoma cells were used for determining the neurite outgrowth. N2a cells were transfected with different constructs using lipofectamine 2000 (Invitrogen), and subjected to differentiation using culture medium with 10 μM retinoic acid (Sigma) and 2% fetal bovine serum (Hyclone). These constructs include HTT19Q (a construct for the control of HD), HTT84Q (a construct for the control of HD), miR-196a and miR-NC. HTT19Q and HTT84Q contain the exon 1 region of the *Huntingtin* gene with 19 or 84 CAG repeats, respectively, under control of a human ubiquitin promoter. These two constructs also carry a *green fluorescence protein (GFP)* gene for further observation. The miR-196a contains the precursor hsa-miR-196a-2 (accession number: MI0000279) under control of a human ubiquitin promoter and a *red fluorescence protein (RFP)* gene for observation. miR-NC is a non-relative control of miRNA. Three days after differentiation, the images of neurite outgrowth were captured under a DM2500 fluorescent microscope (Leica), and analyzed using the Neurite Outgrowth Application Module of a MetaMorph software.

### Statistical analysis

Data were expressed as means ± standard deviations, and Student's t-test was used to compare differences between different groups in the study of neurite outgrowth. Statistical significance was set at P < 0.05.

## Results

In order to compare the expression profiling between HD transgenic mice (GHD) and HD transgenic mice overexpressing miR-196a (D-Tg), we analyzed our previously published microarray data (GSE47500) [14]. This high-throughput data was performed using striatal tissue obtained from the above two age-matched transgenic mouse lines. Various bioinformatic tools were used to further analyze this data, including “the Database for Annotation, Visualization and Integrated Discovery (DAVID)”, “Molecular Signatures Database (MSigDB)”, “TargetScan” and “MetaCore”. DAVID and MSigDB are two widely used repositories providing the biological meaning behind annotated gene sets, while MetaCore is an integrated software package designed for pathway analyses. DAVID was first used to survey categorical data for Gene Ontology (GO), and then the data was imported into MSigDB to narrow down the miR-196a related biological processes. Additionally, TargetScan was used to identify the potential targets of miR-196a, and MetaCore was used to mine the potential pathways further correlating with the miR-196a target genes acquired from TargetScan.

### DAVID

**Fig 1:**
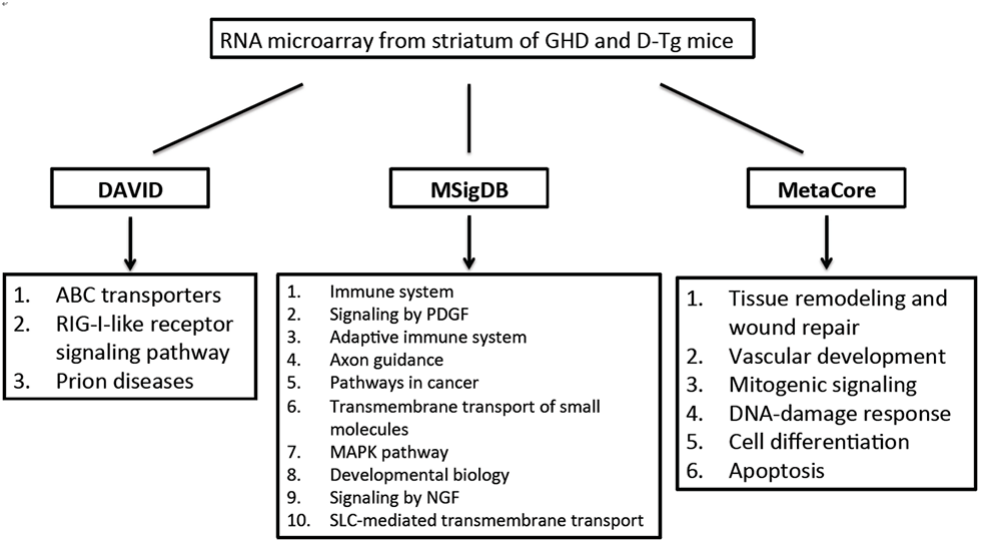
Summary of results from the analyses of different bioinformatic tools. Microarray data (GSE47500) was used to compare the expression profiling between HD transgenic mice (GHD) and HD transgenic mice overexpressing miR-196a (D-Tg). The potential regulatory pathways are listed via different analyses, including DAVID, MSigDB and MetaCore.

Gene Ontology (GO) term analysis was performed with DAVID 6.7 using the functional annotation clustering method [17]. Functional enrichment analysis was applied on the genes in the network with a threshold of *P* < 0.05 from GSE47500. The biological processes highly correlated with miR196a-regulated genes are listed in Fig 1. In short, the first process is “ATP-binding cassette (ABC) transporters” with the most significance, and the 2^nd^ is “retinoic acid-inducible gene 1 (RIG-I)-like receptor signaling pathway” while the 3^rd^ enriched pathway is “prion diseases”. The affected genes involved in the top three pathways are listed in Table 1.

**Table 1:** Gene ontology (GO) analysis of microarray in DAVID.

Category	Term	Count of DEGs	P-value	Genes
KEGG pathway	ABC transporters	8	0.0063	ABCB9, ABCB1A, TAP1, ABCC10, ABCA1, ABCG1, ABCG2, ABCA5
KEGG pathway	RIG-I-like receptor signaling pathway	9	0.0189	IFIH1, DDX3X, IRF7, RIPK1, DDX3Y, NLRX1, AZI2, TRADD, PIN1
KEGG pathway	Prion diseases	6	0.0282	C1QA, C1QB, C8B, NOTCH1, CCL5, C1QC

1094 significant genes (*P* < 0.05) from the microarray were imported into DAVID. GO term analysis was performed to identify biological processes enriched among miR-196a regulated genes (*P* < 0.05). The top three annotation clusters are shown. Category classifications are based on the KEGG database, which is a collection of biological pathways from “Kyoto Encyclopedia of Genes and Genomes”. DEG: differentially expressed gene.

### MSigDB

As MSigDB contains large numbers of gene sets for biologically regulatory pathways, it is one of the most widely used bioinformatic tools [18]. We thus chose MSigDB 4.0 as another approach to identify the possible biological pathways regulated by miR-196a in HD. 1094 genes with significant alteration (p<0.05) from microarray data were imported into MSigDB version 4.0 available from the Gene Set Enrichment Analysis (GSEA) website (http://www.broadinstitute.org/gsea/index.jsp), and the top 10 clustered pathways with the highest scores were obtained. The most influenced pathways include “immune system”, “signaling by platelet-derived growth factor (PDGF)”, “adaptive immune system”, “axon guidance”, “pathways in cancer”, “transmembrane transport of small molecules”, “mitogen-activated protein kinases (MAPK) pathway”, “developmental biology”, “signaling by nerve growth factor (NGF)”, and “SLC-mediated transmembrane transport” (Fig 1).

### MetaCore

**Fig 2:**
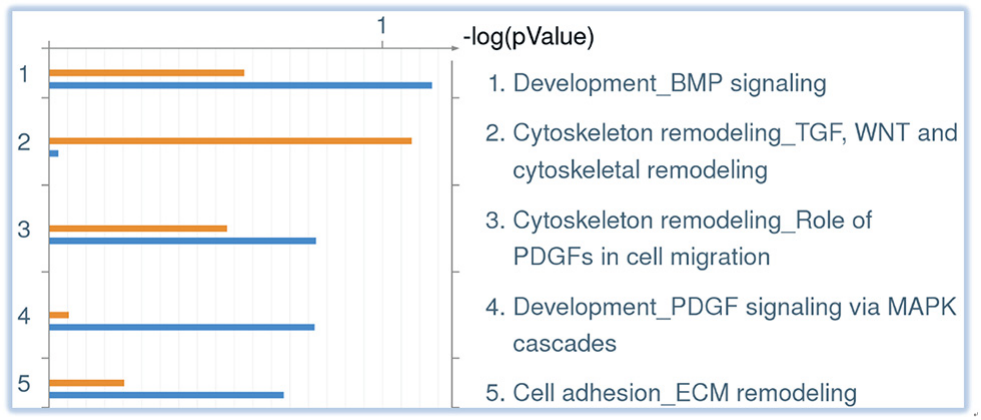
The top five pathways involved in “tissue remodeling and wound repair” via MetaCore analyses. The orange bar stands for significant genes (*P* < 0.05) acquired from microarray data, while the blue bar is the expected gene directly interacting with miR-196 from TargetScan. The list of possible affected pathways is arranged as per descending *P* value score.

Based on the analyses of altered genes via DAVID and MSigDB systems, the specific regulatory pathways affected by miR-196a in HD were predicted. To further narrow down the most likely pathways, the 239 direct target genes that were predicted by the TargetScan 6.2 website were uploaded to a MetaCore tool (GeneGo, USA), and then were analyzed with our microarray dataset as described above. The top six pathway maps are presented in Fig 1. In brief, the downstream targets of miR-196a not only predominantly affect “tissue remodeling and wound repair” but also influence “vascular development”, “mitogenic signaling”, “DNA-damage response”, “cell differentiation” and “apoptosis”. We further analyzed the top five pathways involved in “tissue remodeling and wound repair”, and the results showed that these are “Development-BMP signaling”, “Cytoskeleton remodeling-TGF, WNT and cytoskeleton remodeling”, “Cytoskeleton remodeling-Role of PDGFs in cell migration”, “Development-PDGF signaling via MAPK cascades” and “Cell adhesion-ECM remodeling” as shown in Fig 2.

### The effects of miR-196a on HD related pathways

**Fig 3:**
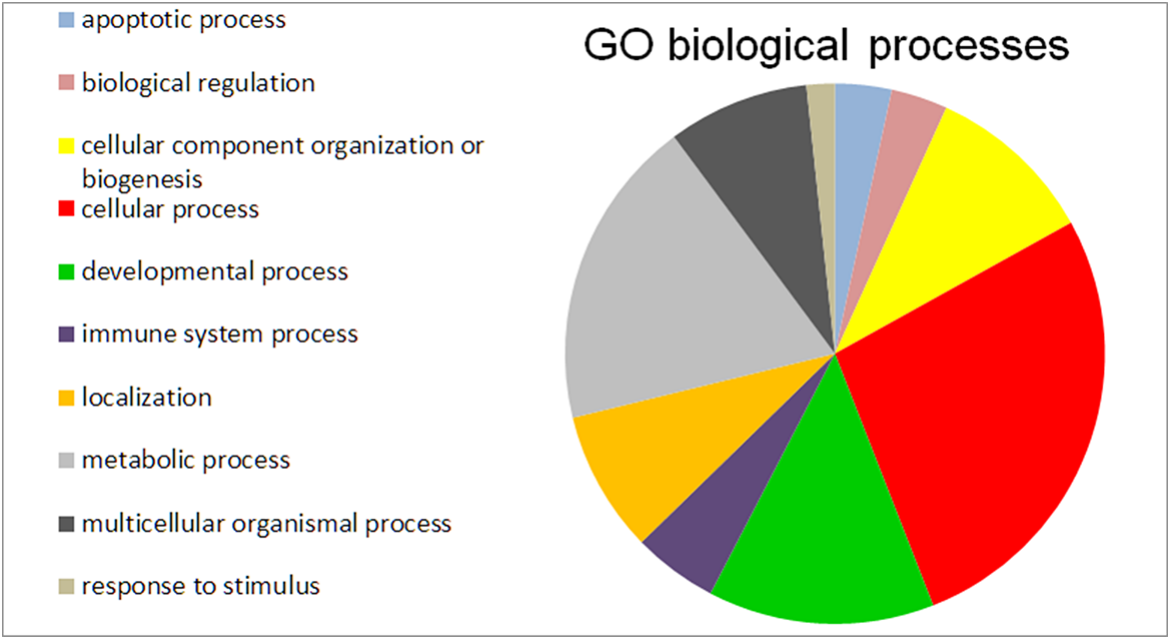
Gene ontology (GO) analysis of forty HD related genes identified from MetaCore. 1094 significant genes were analyzed using the MetaCore built-in HD pathway, and 40 HD-related genes were identified. These 40 genes were subjected to GO analysis, and the top ten GO biological processes are listed and shown in the pie chart.

Since the phenotypes of HD were alleviated by miR-196a in our previous study[14], in the current work we are attempting to comprehensively analyze the effects of miR-196a on HD-related pathways. Again, we input 1094 genes from our microarray data into MetaCore, and analyzed these genes using the HD specific pathway built in this bioinformatics tool. The results showed there are 40 significantly altered genes involved in the HD pathway as listed in Table 2. In addition, we not only analyzed the HD specific pathway, but also examined 1094 genes in other HD-related pathways using the MetaCore built-in data base. We found these genes are highly involved in the “apoptosis and survival pathway”, “cytoskeleton remodeling”, “cell adhesion”, and so on (Table 3). We further subjected the 40 genes identified above to Gene Ontology analysis, and found that these are dominantly involved in the “cellular process”, “metabolic process” and “develop process” (Fig 3). These results suggest miR-196a does alter several critical pathways related to HD.

**Table 2:** Forty altered genes related to HD from the microarray data.

Gene Symbol (#1-#10)	miR-196a + HD/ HD	Gene Symbol (#11–20)	miR-196a + HD/ HD	Gene Symbol (#21-#30)	miR-196a + HD/ HD	Gene Symbol (#31-#40)	miR-196a + HD/ HD
**ATF6B**	1.2017845	**GABBR1**	1.1112367	**GRID1**	-1.2644141	**PRDX5**	2.0336182
**ADORA2A**	1.3786301	**GFAP**	-1.7528172	**PDYN**	1.307083	**PRL**	-1.7513222
**CASP1**	-1.2626195	**CNR2**	-1.2639676	**MAOA**	-1.1514896	**RAB3C**	1.2916043
**CNR2**	-1.2639676	**ACKR2**	1.2096476	**MAOA**	-1.1514896	**SGK1**	-1.1918408
**COX7B2**	-1.2578122	**ADORA2A**	1.3786301	**NAPB**	1.2149822	**C8orf44-SGK3**	-1.1967563
**COX7B2**	-1.2578122	**ACKR2**	1.2096476	**NDUFS1**	-1.1015196	**NAPB**	1.2149822
**CASP1**	-1.2626195	**ADORA2A**	1.3786301	**NDUFV3**	-1.2432787	**STX1A**	-1.2138777
**DNM1L**	-1.1338472	**PRPF40B**	1.2933092	**PRDX6**	2.0336182	**TCERG1**	-1.1931573
**GABRB2**	1.2547606	**HIST1H2AC**	1.2534748	**PDE6G**	1.2838423	**JMJD1C**	-1.1645135
**GABRR2**	-1.3007302	**HIST1H2AC**	1.2534748	**PRDX6**	2.0336182	**TXNRD1**	-1.085796

1094 significant genes were analyzed through the MetaCore built-in HD pathway, and 40 HD related genes were identified. Gene symbols and expression fold-changes are listed.

**Table 3:** Enrichment analysis by pathway maps in the folder 'Huntington Disease' of MetaCore.

#	Pathways	Total	In Data	Network Objects from Active Data
1	Apoptosis and survival: Endoplasmic reticulum stress response pathway	55	6	Derlin-3, EDEM, eIF2AK3, DNAJC3, PP1-cat, ERP5
2	Cytoskeleton remodeling: Neurofilaments	25	3	Tubulin beta, GFAP, Vimentin
3	Cell adhesion: ECM remodeling	52	5	Collagen IV, PLAT (TPA), MSN (moesin), MMP-16, Kallikrein 2
4	Oxidative stress Role of Sirtuin1 and PGC1-alpha in activation of antioxidant defense system	60	5	TXNRD2, PRDX5, GSTA5, TXNRD1, GCL reg
5	Immune response: MIF—the neuroendocrine-macrophage connector	46	4	ABCA1, Galpha(s)-specific CRF GPCRs, CD74, PLA2
6	Transcription: CREB pathway	49	4	MSK1/2 (RPS6KA5/4), Proenkephalin-B, PP1-cat, Rac1
7	Apoptosis and survival: Caspase cascade	34	3	TRADD, Caspase-6, RIPK1

1094 significant genes were analyzed through the MetaCore built-in HD related pathways, and seven critical pathways are listed. “Total” indicates the total number of genes involved in different specific pathways of the MetaCore database. “In Data” indicates the number of significantly altered genes matching the genes in those specific pathways.

### Neurite outgrowth

**Fig 4:**
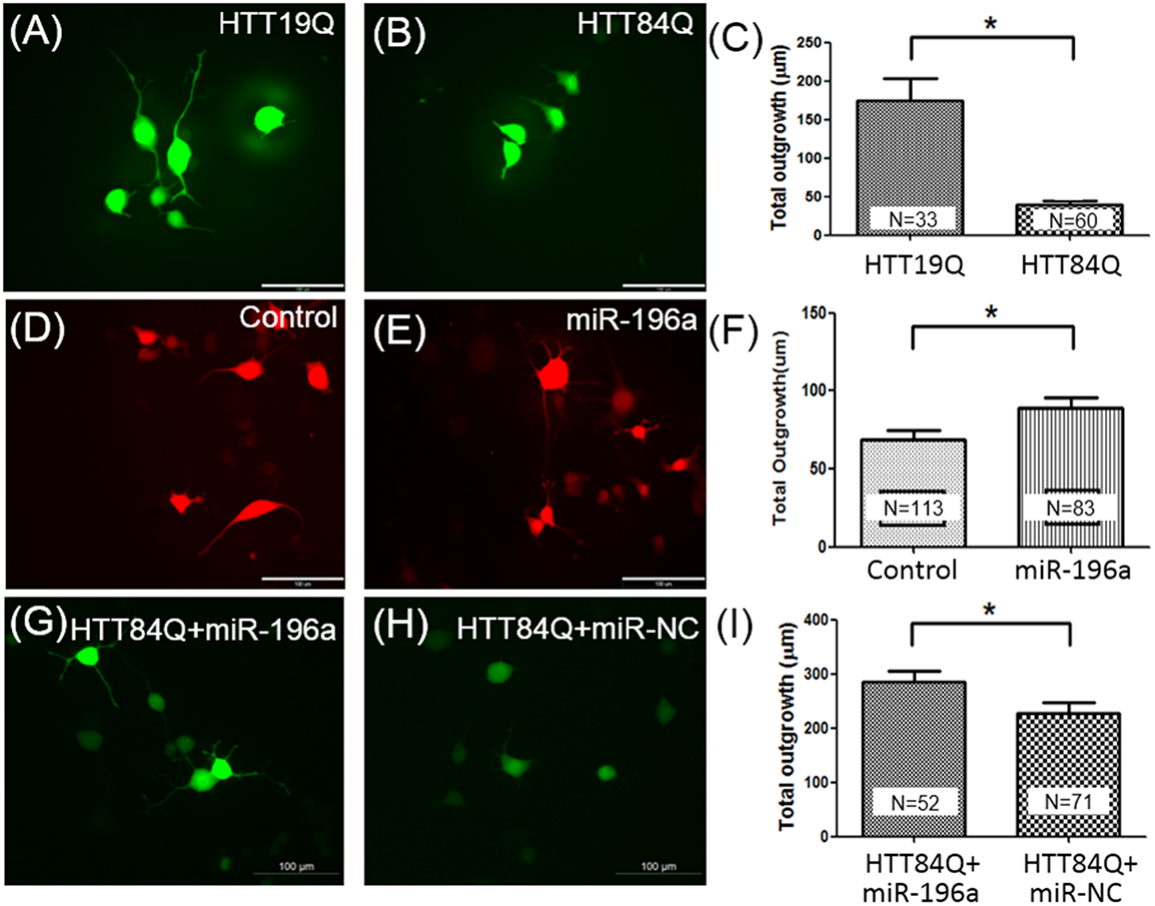
The effects of miR-196a on neurite outgrowth in N2a mouse neuroblastoma cells. N2a cell were transfected with different constructs, including HTT19Q (A), HTT84Q (B), miR-non relative control (NC; D), miR-196a (E), HTT84Q+miR-196a (G) or HTT84Q+miR-NC (H), and neurite outgrowth was analyzed though the Neurite Outgrowth Application Module of the MetaMorph software. Fluorescent images(A, B, D, E, G and H) show the morphology of neurite outgrowth in different groups as indicated, and statistically quantitated data between HTT19Q and HTT84Q (C), Control and miR-196a (F) and HTT84Q + miR-196a and HTT84Q + miR-NC (I) are shown.

Based on above results of the above analyses, we speculate that miR-196a might enhance the neuronal cytoskeleton to improve pathological phenotypes in HD. Since the neuronal skeleton is related to the development of neuronal cells, we next examine the neurite outgrowth in neuron-like cells. We first determine the neurite outgrowth of N2a neuroblastoma cells under the HD condition. As we transfected HTT19Q (a control group) and HTT84Q (a HD group) plasmids into N2a cells, the total neurite outgrowth was significantly shorter in the HTT84Q group compared to that of the HTT19Q group (Fig 4A–4C; P<0.05), suggesting less neurite outgrowth under the HD condition. We further studied the effects of miR-196a on neurite outgrowth, and found the miR-196a could significantly enhance neurite outgrowth compared to the control group (Fig 4D–4F; P<0.05). Furthermore, we also examined the effects of miR-196a on neurite outgrowth under the HD condition, and the results showed that miR-196a significantly increased this compared to the results obtained with the non-relative control (Fig 4G–4I; P<0.05). These results help to validate some of the bioinformatic analyses, and suggest that miR-196a might provide protective effects with regard to HD through enhancement of the cytoskeleton.

## Discussion

Bioinformatic tools have accelerated the progression of biomedical research, with high-throughput screening being especially useful. Such tools not only reduce the need for time-consuming and labor-intensive bench-work, but also provide critical directions and insights for advanced studies. In our previous studies, we showed that miR-196a could improve the neuropathological phenotypes in different models of HD [14]. We further performed high-throughput studies by using striatum of transgenic mice, and identified 1094 genes with significant alterations. Using analyses of DAVID, MSigDB and MetaCore, we organized the possible regulatory pathways, such as those of “ABC transporters”, “RIG-I-like receptor signaling”, “immune system”, “issue remodeling and wound repair”, and so on, controlled by miR-196a in HD, as shown in Fig 1. These results show the possible effects of miR-196a on HD, and it is anticipated that they will help in the development of therapeutic strategies to treating HD.

GO term analyses using DAVID showed “ABC transporters” and “RIG-I-like receptor signaling pathway” are the top two clustered biological processes which may be involved in the beneficial effects of miR-196a on HD. Based on previous studies, ABC proteins are responsible for transporting various substrates across biological membranes, including cholesterol [19]. Cholesterol plays an important role in neurite outgrowth, and cholesterol synthesis has been reported to be dysfunctional in HD [20, 21]. Cholesterol is also one of the critical compositions of myelin. This thus suggests that abnormal ABC transporters may interfere with cholesterol transportation, and further lead to demyelination in HD. Furthermore, ABC transporters are altered in several neuronal diseases, such as traumatic brain injury, amyotrophic lateral sclerosis, Parkinson's disease and Alzheimer's disease [22–26]. Due to the similarities among several different neurodegenerative diseases, ABC transporters may influence neurite outgrowth through lipid transportation in both HD and other neurodegenerative diseases. One direction for future studies is thus to examine this regulatory pathway in HD.

Another enriched regulatory pathway, the RIG-I-like receptor signaling pathway, takes part in ubiquitin-mediated proteolysis and MAPK signaling[27, 28]. In addition, the data from MSigDB also shows alteration of the “MAPK pathway”(Fig 1). Since both the ubiquitin-proteasome system and MAPK signaling are highly involved during HD pathogenesis, this suggests that RIG-I may be an important upstream regulator contributing to the effects of miR-196a in HD. Moreover, RIG-I associates with actin, a fundamental component of the cytoskeleton [29], suggesting miR-196a may also regulate the neuronal structure through not only ABC transporters, but also RIG-I-like receptor signaling pathways. The results of GO term analyses using DAVID thus strongly suggest that miR-196a might enhance the neuronal skeleton to improve pathological phenotypes in HD.

The enriched pathways derived from the MSigDB data sets revealed that the “immune system” and “adaptive immune system” are two critical pathways involved in this alteration. A number of scientists recently examined the immune system in relation to HD, and also found that both innate and adaptive immune systems are activated during the progression of this disease [30–33]. More specifically, mutant huntingtin leads to a migration deficit of immune cells through defective actin remodeling [34]. Putting these results together also suggests that miR-196a might affect the cytoskeleton, and thus have beneficial effects in HD.

The data from MetaCore show that the “tissue remodeling and wound repair” pathway is predominantly affected (Fig 1). Tracing the possible mechanisms comprised in this category shows that “cytoskeleton remodeling” plays the most critical role (Fig 2). According to prior studies, the cytoskeletal is a key factor contributing to the pathogenesis of HD [35–37]. In addition, several cytoskeletal associated proteins, such as huntingtin-associated protein-1 (HAP-1), huntingtin-interacting protein-1 (HIP1), and tubulin, have been confirmed to interact with HTT [38–40]. In addition, our examination of neurite outgrowth revealed that this was also enhanced after an increase in miR-196a (Fig 4). These results are not only consistent with the analyses of DAVID and MSigDB, but also show the important role of the cytoskeleton with regard to the beneficial functions that miR-196a has in HD. In particular, most neuronal diseases are highly related to the functions of the cytoskeleton, suggesting one potential direction for investigating the working mechanisms of miR-196a.

The phenotypes of HD were alleviated by miR-196a in our previous study[14], and in current work we showed that several genes and critical pathways related to HD were also altered in our microarray data (Tables 2 and 3; Fig 3). In particular, cytoskeleton remodeling related to HD is involved in the effects of miR-196a as described above (Table 3). Apoptosis, caspase cascade, CREB pathway, metabolic process, and so on are all well-studied during the progression of HD [1], and have been identified in this study. These results strongly suggest that miR-196a not only improves the pathological and behavioral phenotypes, but also works through these well-defined pathways of gene regulation in HD.

In sum, we analyzed the microarray data to compare the differential profiling of gene expression in HD transgenic mice with or without miR-196a overexpression via three different bioinformatics tools. The results showed that miR-196a has the potential to provide neuroprotective functions through regulation of the cytoskeleton in HD. Since several studies have addressed the effects of miR-196a on neuronal diseases [41, 42], it is anticipated that the results of this analysis based on high throughput screening will provide insights that can aid in the development for therapeutic strategies for treating HD.
